# The Interplay Between Cortical State and Perceptual Learning: A Focused Review

**DOI:** 10.3389/fnsys.2018.00047

**Published:** 2018-10-09

**Authors:** Sung Eun Kwon

**Affiliations:** Department of Molecular, Cellular and Developmental Biology, University of Michigan, Ann Arbor, MI, United States

**Keywords:** brain state, population activity, perceptual learning, attention, sensory processing

## Abstract

Measurements of population activity in alert animals have demonstrated that the intrinsic response state of the cortex has profound effects on the neuronal representation of sensory inputs, raising the possibility that cortical state could influence the behavioral performance in perceptual learning (PL). PL is a process by which sensory experience leads to gradual and semi-permanent improvements in perceptual judgment, and it is generally agreed that these improvements are modulated by sensory cortical areas. Although the precise neural mechanisms underlying the improved perceptual judgment remain unclear, cortical state has been shown to impact the behavioral outcome of PL. We discuss several ways in which cortical state might influence PL based on the recent evidence for state-dependent modulation of sensory encoding. Conversely, training in a certain perceptual task feeds back to modulate cortical state, suggesting a bi-directional relationship between cortical state and behavioral outcomes of PL. We highlight the recent studies that shed light on the mechanism of the interplay between cortical state and PL.

## Introduction

**FIGURE 1:**
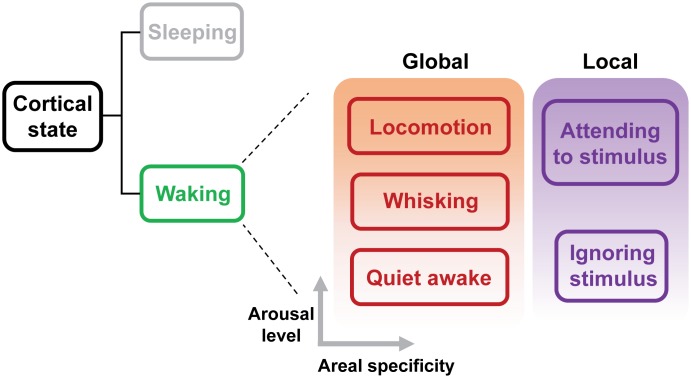
Global and local substates of wakefulness. Sleep and wakefulness are the two fundamental cortical states. Recent studies have revealed that, even during wakefulness, the cortical state exhibits transitions between different sub-states characterized as different levels of arousal (Gervasoni et al., 2004; Harris and Thiele, 2011; McGinley et al., 2015b). Substate transitions may occur globally or within specific areas depending on behavioral demands. A hallmark of a globally aroused state such as locomotion is cortical desynchronization. Selective attention leads to a local state transition, which manifests as the desynchronization in cortical areas responsive to the attended stimuli. Therefore, both global and local state transitions appear to use similar mechanisms. Advances in recording techniques and behavioral approaches will provide an increasingly more elaborate taxonomy of cortical state.

Cortical state classically refers to the dynamics of neuronal population activity in a cortical network (Harris and Thiele, 2011). Transitions of cortical states have been typically described as changes of spontaneous ongoing network activity. Early studies using electroencephalogram (EEG) and local field potential (LFP) recordings have revealed two fundamental states that exist in the neocortex: a synchronized state characterized by large-amplitude, low-frequency (0.2–10 Hz) spontaneous fluctuations between the up and down phases, and a desynchronized state characterized by small-amplitude, high-frequency (25–100 Hz) fluctuations whereby neighboring neurons spike more independently (Steriade et al., 2001). Conventionally, the synchronized state has been associated with sleeping or anesthetized brain, and the desynchronized state with alert brain (Steriade et al., 2001). The transition between these two activity states plays a central role in modulating sensory-evoked population activity in the cortex. Recent studies in head-fixed awake rodents have demonstrated that waking state actually consists of distinct “substates,” including a state similar to the synchronized state usually observed during sleep (**Figure 1**; Poulet and Petersen, 2008; Sachidhanandam et al., 2013; Reimer et al., 2014; Tan et al., 2014; McGinley et al., 2015a,b; Vinck et al., 2015). These substates exhibit rapid and spontaneous transitions, which reflect global fluctuation in the levels of arousal and movement, and have a major impact on sensory processing and behavioral performance. Another line of investigation has been focused on the local changes in cortical states during tasks that involve selective attention, studied in non-human primates (Desimone and Duncan, 1995; Fries et al., 2001; Cohen and Maunsell, 2009; Gregoriou et al., 2009; Mitchell et al., 2009; Chalk et al., 2010). Shifts in local cortical state during selective attention appear to have similar effects on sensory encoding to those elicited by the global state changes. Both selective attention and global arousal enhance the representation of sensory information by increasing evoked firing rate, decreasing response variability and reducing correlated noise (Luck et al., 1997; Cohen and Maunsell, 2009; Mitchell et al., 2009; Reimer et al., 2014; Vinck et al., 2015).This suggests that the effects of both global and local state transitions might be mediated by overlapping mechanisms.

How these cortical states are orchestrated to modulate neural and behavioral plasticity during learning is a fundamental question in neuroscience. Recent findings of functional MRI (fMRI) studies in human subjects show that cortical state, measured as the resting state network activity of the brain has a significant impact on the performance in perceptual learning (PL) (Baldassarre et al., 2012; Freyer et al., 2013). Here, we review how cortical state has been defined in the latest studies, highlight ways in which cortical state may influence PL and vice-versa, and discuss an integrative framework that could guide future investigation.

## Definitions of Cortical State in the Awake Brain

Cortical state has been defined operationally on different spatial scales (from a specific area to the entire cortex) and temporal scales (tens of milliseconds to seconds) even in the awake brain. At the neuronal level, shifts in cortical state manifest as a transition between up (depolarized) and down (hyperpolarized) phases of membrane potential, which arises from fluctuations in the synaptic input. Cortical state has also been defined based on population-level fluctuations in synchrony or the LFP power during spontaneous activity (Harris and Thiele, 2011). Simultaneous measurements of membrane potential, LFP and behavioral state (e.g., movement, muscle tone, pupil diameter) in head-fixed mice have revealed that neuronal and brain-wide state fluctuations are generally correlated and that pupil dilation is indicative of desynchronized or “aroused” state (Reimer et al., 2014; McGinley et al., 2015a; Vinck et al., 2015). Cortical state monitored in these studies is associated with fluctuations in the global behavioral state such as arousal and locomotion. In studies that used ensemble spiking activity recording, cortical state has been defined based on fluctuations of the synchrony (Luczak et al., 2013; Pachitariu et al., 2015; Scholvinck et al., 2015) and the strength (Arandia-Romero et al., 2016; Engel et al., 2016; Beaman et al., 2017; Gutnisky et al., 2017) of population activity in a local cortical area. Attempts have been made to correlate trial-to-trial fluctuation of spontaneous and evoked population activity to sensory encoding and behavioral responses. This line of work shows that cortical state can exhibit rapid trial-to-trial shifts, modulate sensory encoding and impact perceptual behavior (Arandia-Romero et al., 2016; Engel et al., 2016; Beaman et al., 2017; Gutnisky et al., 2017). Despite the differences in experimental settings and ways in which cortical state was defined (e.g., synchrony-based vs. strength-based definitions), these studies agree on a general point: certain patterns of ongoing network activity reflecting a cortical state enhance sensory encoding, and transitions in cortical state correlate well with fluctuations in encoded sensory information and the behavioral report. Thus, cortical response is not exclusively determined by external stimulus but by the interaction between the sensory stimulus and internal state reflected in the spontaneous population activity. How does cortical state impact learning? Here, we will attempt to answer this question by focusing on PL, in which sensory cortex has been shown to play an important role (Caras and Sanes, 2017).

## Cortical State and Perceptual Learning

Perceptual learning is a form of implicit learning and distinct from declarative learning that requires the use of the medial temporal lobe (Gilbert and Sigman, 2007; Watanabe and Sasaki, 2015). PL is characterized by a gradual, long-term improvement in perceptual judgement. Neural plasticity mechanisms associated with PL are thought to be specific to the trained stimulus and task. However, recent studies demonstrated that perceptual improvement in one task can transfer to other tasks (Xiao et al., 2008; Zhang et al., 2010). Therefore it is still under debate whether and under what circumstances PL generalizes to other tasks and sensory features. Many studies have reported cortical changes associated with PL, including prominent map expansion, increases in receptive field size, sharpening and amplification of tuning curves to the trained feature and reduction of correlated variability among neurons in early sensory cortex (Recanzone et al., 1993; Schoups et al., 2001; Ni et al., 2018). However, these changes are not always observed in early sensory cortex (Ghose et al., 2002) but rather in the intermediate sensory cortex or the sensory-motor area (Yang and Maunsell, 2004; Law and Gold, 2008) and do not necessarily correlate with performance improvement. Moreover, the involvement of early sensory cortex in itself does not explain the context-specificity of PL. Performance improvement within one task does not transfer to another task that uses the same set of stimuli in a different context, suggesting that the outcome of PL is under “top-down” influence (Ahissar and Hochstein, 1993; Gilbert and Sigman, 2007).

Neural mechanisms that underlie behavioral improvement associated with PL remain undefined. Depending on the task design, species and sensory modality, different mechanisms have been put forward. In tasks that require discrimination or detection of primary features of stimulus, the performance improvement has been associated with enhanced representation of the relevant features. On the other hand, tasks that are based on emergent properties of cortex such as context-specific sensory coding, remarkable changes in the top-down influence have been observed while the neural plasticity at the level of individual sensory neurons appeared to be modest (Crist et al., 2001). Another potential mechanism for the neural plasticity during PL involves reweighting connections between sensory and decision cortical areas via trial-by-trial feedback signals (Dosher and Lu, 1998; Seitz and Watanabe, 2005; Law and Gold, 2009). This leads to an improved “read-out” of sensory evidence by a decision unit and a reduced internal noise (Dosher and Lu, 1998). A similar model applied to feedforward connections between thalamus and early sensory cortex also captures behavioral improvements during PL (Bejjanki et al., 2011). Therefore cortical state may impact PL via enhancing the representation of task-relevant sensory input, improving context-dependent recruitment of relevant circuits and reducing internal noise. In the following section, we will discuss the impact of cortical state on sensory encoding as a potential mechanism for PL. Importantly cortical state is subject to various forms of modulation. We will review the evidence supporting that top-down feedback signals from “higher” cortical areas modulate cortical state and how such modulation might occur during PL (Gilbert and Sigman, 2007; Law and Gold, 2009).

## The Impact of Cortical State on Stimulus Encoding

An intuitive way by which cortical state might affect PL is through modulating neural activity in the cortical area that encodes task-relevant features. Typical PL involves training subjects to discriminate perceptually demanding stimuli that are repeatedly presented for extended periods of time. Neuronal responses to repetitive sensory stimuli are not static but dynamically modulated by the ongoing spontaneous activity. Recent studies using population recording techniques have revealed that spontaneous activity is structured in spatial and temporal domains. The amplitude and variability of sensory-evoked responses are correlated with the intrinsic response state in the sensory cortex to such an extent that the evoked responses can be predicted from the pre-stimulus on-going activity (Curto et al., 2009; Scholvinck et al., 2015). Stimulus statistics is another factor that affects the internal cortical state during the presentation of a sensory input. For example, unexpected brief stimuli evoke robust responses regardless of cortical states, although the response amplitude is smaller in desynchronized states (Castro-Alamancos, 2004; Otazu et al., 2009). Responses to stimuli with high repetition frequencies show a more complex state-dependence. While the response to the first stimulus in a train with a high repetition frequency is elevated in synchronized states compared to desynchronized states, responses adapt more strongly in a synchronized state (Castro-Alamancos, 2004). These observations suggest that sensory stimuli presented with repetition may lead to enhanced neuronal representation in a desynchronized state. Across modalities, temporally extended stimuli are more faithfully represented in desynchronized than synchronized states, which might be beneficial for animals with behavioral needs to analyze fine details of continuous sensory inputs (Goard and Dan, 2009; Marguet and Harris, 2011).

State-dependent improvement in sensory encoding can be observed at the population level. Network-level changes have been measured recently using population recording techniques, which demonstrated that the amount of sensory information encoded in population activity was significantly higher in desynchronized states (Pachitariu et al., 2015; Beaman et al., 2017). A potential explanation for the improved population decoding in these studies is a decrease in the correlated variability among neuronal population during desynchronized state. *In vivo* measurements in different cortical areas show that the pairwise correlated variability is on average about 0.1–0.2, although it can vary significantly depending on internal cortical state (Cohen and Kohn, 2011; Ecker et al., 2014). Recurrent network models of anatomically inspired connectivity yield a similar value (Shadlen et al., 1996). This correlated variability severely limits the amount of information encoded by neuronal population as it cannot be removed by simply pooling responses across neurons. Although the relationship between cortical state and the correlated variability is still under debate, many studies have observed that the correlated variability is reduced in a desynchronized state, which would lead to improved population coding (Pachitariu et al., 2015; Scholvinck et al., 2015; Gutnisky et al., 2017). An elegant study has revealed that the reduction in correlated variability is a common mechanism for improved performance during PL and attention, although these processes occur on different time scales (Ni et al., 2018).

Circuit mechanisms by which state-dependent changes in pairwise correlations are implemented during PL remain poorly understood. Globally desynchronized states, such as arousal and locomotion, enhance sensory-evoked responses and reduce the correlated noise among neurons, leading to increases in encoded sensory information. However, such global states do not explain the neural changes that occur locally within relevant brain areas during some PL tasks. Moreover, it is unclear whether desynchronization leads directly to decorrelation, because the relationship between spiking synchrony and the correlated variability is not straightforward. It is possible to have a decorrelated cortical network with two highly synchronized groups of neurons if the two populations cancel each other out, as would be in the case of populations of excitatory and inhibitory neurons (Harris and Thiele, 2011). Indeed, theoretical models support that inhibition might play a critical role in active decorrelation of cortical neurons (Renart et al., 2010; Tetzlaff et al., 2012; Kanashiro et al., 2017). Long-range cortico-cortical and neuromodulatory projections have been shown to provide synaptic inputs to local inhibitory interneurons in target sensory cortical areas (Letzkus et al., 2011; Lee et al., 2013; Pi et al., 2013; Zhang et al., 2014). These interneurons are likely to be recruited in PL, and may serve to mediate state-dependent modulation of neuronal correlations during this learning process. Accumulating experimental evidence supports the roles of the interneurons in different forms of learning. Whether inhibitory interneurons are indeed important for decorrelation of cortical network over the course of PL is an important avenue for future investigation.

## Modulation of Cortical State by Perceptual Learning

As we explore our surroundings, we are constantly bombarded with sensory stimuli. Depending on the behavioral context and outcome, some sensory stimuli are filtered out while others are registered for further processing, suggesting that cortical state is not static but dynamically modulated by ongoing behavioral demands. While, as discussed above, certain cortical states have been shown to enhance sensory encoding and facilitate PL, it is important to note that PL in turn modulates cortical state in a context-specific manner. How does the brain assess behavioral context and update cortical state accordingly? To understand the context-dependent modulation of cortical state during PL, it would be necessary to determine at what time point in a trial such modulation occurs in relation to the switching of the context. In a pioneering study, animals were trained for two different visual PL tasks that involved the same set of stimuli (Li et al., 2004). It is clear from this work that when cued to the task to be performed before the stimulus onset, the difference in the response arises at the outset of the neurons’ responses, indicating that the top-down information conveying the task instruction sets the cortex to a state that then enables analysis of the stimulus in a task-relevant fashion. In a recent study, the cortical state defined by the population activity during a delay period prior to the presentation of the test stimulus, switches to a “low activity” mode, and this shift in state showed trial-by-trial correlation with encoded sensory information and behavioral performance in an orientation discrimination task (Gutnisky et al., 2017). These studies suggest that signals carrying information about behavioral outcome propagate back to early sensory cortex, and that this “top-down” influence (e.g., expectation or prediction) sets cortical state in preparation for the ongoing behavioral demand. Studies using two-photon calcium imaging have revealed diverse sources of long-range projections to early sensory cortex, including those from higher cortical areas, higher thalamic nuclei and amygdala, which carry information about context, behavioral outcome and physiologically relevant sensory cues (Makino and Komiyama, 2015; Burgess et al., 2016; Kwon et al., 2016; Roth et al., 2016).

A widely studied instance of top-down influence is spatial attention. In the majority of PL tasks that involve active engagement of subjects, attention is required for behavioral improvement. It is generally agreed that attention enhances performance in perceptual tasks by increasing the neuronal gain and reducing the correlated noise shared among similarly tuned neurons, thereby leading to improved sensory coding and enhanced performance (Cohen and Maunsell, 2009; Rabinowitz et al., 2015). It has also been reported that attention modulates network oscillation. Attention decreases low frequency fluctuation while increasing the fluctuation at gamma frequencies (25–100 Hz) in task-relevant brain areas, reflecting local cortical desynchronization (Fries et al., 2001; Gregoriou et al., 2009; Mitchell et al., 2009). A recent study investigated changes in synchronous spiking in a visual cortex during a visual task that required spatial attention (Engel et al., 2016). By modeling the transition between vigorous and faint spiking states, this study demonstrated that the impacts of spatial attention on firing rate and the transition dynamics can be separated. Importantly, the local modulation of the state transition predicted the behavioral performance in the task.

These observations suggest that various forms of the top-down influence set cortical state into a mode that enables efficient sensory processing and perceptual improvement through desynchronizing cortical network and reducing the correlated variability. As a potential mechanism of top-down influence, enhanced selective attention during PL modulates cortical state such that population coding of relevant sensory input is improved, which leads to behavioral improvements (Byers and Serences, 2014; Ni et al., 2018). In addition to this “permissive” role, it is possible that some cortical states may play a causal role by actively mediating or driving the neural plasticity underlying PL. Theoretical works suggest that shifts in network oscillations such as the increase in gamma power might organize neurons into a cell assembly that is capable of registering and evaluating sensory inputs during an ongoing task (Borgers et al., 2008). The increase in gamma power has been shown to correlate with enhanced coupling between cortical areas during attention (Gregoriou et al., 2009). Therefore, it still remains to be determined whether cortical state plays a direct causal role or merely allows performance improvements during PL. In order to establish the necessity of a certain cortical state for behavioral outcome of PL, one needs to manipulate cortical state in behaving subjects and test its effect on sensory encoding and performance during PL. This is an important area of ongoing and future investigation.

## Future Outlook

A tremendous progress has been made in our understanding of cortical state and how cortical state and PL impact each other. However, there are important gaps to be filled. While recent studies have revealed fine spatiotemporal structures in cortical state, it remains poorly understood how neurons of different cell-types orchestrate their activity during shifts in cortical state. As discussed above, inhibitory interneurons are thought to be important effectors during cortical state changes. In addition, excitatory neurons in the neocortex have specific projection targets. How are neurons of different cell-types, defined by neurotransmitter and projection patterns, involved in cortical state changes? To fill this important gap, experimental efforts need to be made to measure cell type-specific activity patterns during cortical state transitions.

Many labs have demonstrated a tight correlation between cortical state and sensory processing during various perceptual tasks. More attempts need to be made to establish a causal relationship between changes in cortical state and behavioral changes during PL. Optogenetic manipulation to add or suppress activity during a certain cortical state in behaving animals would be a powerful approach to test the causality of cortical state for sensory processing and perception on a trial-by-trial basis.

It is important to note that PL involves plasticity in several different brain areas. Although it is debatable which brain area(s) causes perceptual improvement, interaction between different cortical areas is likely to be critical for PL. Therefore, studies examining the impact of cortical state on PL and vice versa should consider the long-range pathways that connect different cortical areas. To this end, genetic tools for directly manipulating specific long-range pathways may be combined with recording of cortical state during perceptual tasks. Advances in multi-areal, single-neuron imaging in behaving animals would allow us to define how cortical state transitions are coordinated across brain areas and whether a specific coordination is important for PL.

## Author Contributions

SK wrote the paper.

## Conflict of Interest Statement

The author declares that the research was conducted in the absence of any commercial or financial relationships that could be construed as a potential conflict of interest.

## References

[B1] Ahissar M. Hochstein S. (1993). Attentional control of early perceptual learning. *Proc. Natl. Acad. Sci. U.S.A.* 90 5718–5722. 10.1073/pnas.90.12.5718 8516322 PMC46793

[B2] Arandia-Romero I. Tanabe S. Drugowitsch J. Kohn A. Moreno-Bote R. (2016). Multiplicative and additive modulation of neuronal tuning with population activity affects encoded information. *Neuron* 89 1305–1316. 10.1016/j.neuron.2016.01.044 26924437 PMC5129626

[B3] Baldassarre A. Lewis C. M. Committeri G. Snyder A. Z. Romani G. L. Corbetta M. (2012). Individual variability in functional connectivity predicts performance of a perceptual task. *Proc. Natl. Acad. Sci. U.S.A.* 109 3516–3521. 10.1073/pnas.1113148109 22315406 PMC3295318

[B4] Beaman C. B. Eagleman S. L. Dragoi V. (2017). Sensory coding accuracy and perceptual performance are improved during the desynchronized cortical state. *Nat. Commun.* 8:1308. 10.1038/s41467-017-01030-4 29101393 PMC5670198

[B5] Bejjanki V. R. Beck J. M. Lu Z. L. Pouget A. (2011). Perceptual learning as improved probabilistic inference in early sensory areas. *Nat. Neurosci.* 14 642–648. 10.1038/nn.2796 21460833 PMC3329121

[B6] Borgers C. Epstein S. Kopell N. J. (2008). Gamma oscillations mediate stimulus competition and attentional selection in a cortical network model. *Proc. Natl. Acad. Sci. U.S.A.* 105 18023–18028. 10.1073/pnas.0809511105 19004759 PMC2584712

[B7] Burgess C. R. Ramesh R. N. Sugden A. U. Levandowski K. M. Minnig M. A. Fenselau H. (2016). Hunger-dependent enhancement of food cue responses in mouse postrhinal cortex and lateral amygdala. *Neuron* 91 1154–1169. 10.1016/j.neuron.2016.07.032 27523426 PMC5017916

[B8] Byers A. Serences J. T. (2014). Enhanced attentional gain as a mechanism for generalized perceptual learning in human visual cortex. *J. Neurophysiol.* 112 1217–1227. 10.1152/jn.00353.2014 24920023 PMC4122726

[B9] Caras M. L. Sanes D. H. (2017). Top-down modulation of sensory cortex gates perceptual learning. *Proc. Natl. Acad. Sci. U.S.A.* 114 9972–9977. 10.1073/pnas.1712305114 28847938 PMC5604044

[B10] Castro-Alamancos M. A. (2004). Absence of rapid sensory adaptation in neocortex during information processing states. *Neuron* 41 455–464. 10.1016/S0896-6273(03)00853-5 14766183

[B11] Chalk M. Herrero J. L. Gieselmann M. A. Delicato L. S. Gotthardt S. Thiele A. (2010). Attention reduces stimulus-driven gamma frequency oscillations and spike field coherence in V1. *Neuron* 66 114–125. 10.1016/j.neuron.2010.03.013 20399733 PMC2923752

[B12] Cohen M. R. Kohn A. (2011). Measuring and interpreting neuronal correlations. *Nat. Neurosci.* 14 811–819. 10.1038/nn.2842 21709677 PMC3586814

[B13] Cohen M. R. Maunsell J. H. (2009). Attention improves performance primarily by reducing interneuronal correlations. *Nat. Neurosci.* 12 1594–1600. 10.1038/nn.2439 19915566 PMC2820564

[B14] Crist R. E. Li W. Gilbert C. D. (2001). Learning to see: experience and attention in primary visual cortex. *Nat. Neurosci.* 4 519–525. 10.1038/87470 11319561

[B15] Curto C. Sakata S. Marguet S. Itskov V. Harris K. D. (2009). A simple model of cortical dynamics explains variability and state dependence of sensory responses in urethane-anesthetized auditory cortex. *J. Neurosci.* 29 10600–10612. 10.1523/JNEUROSCI.2053-09.2009 19710313 PMC2861166

[B16] Desimone R. Duncan J. (1995). Neural mechanisms of selective visual attention. *Annu. Rev. Neurosci.* 18 193–222. 10.1146/annurev.ne.18.030195.001205 7605061

[B17] Dosher B. A. Lu Z. L. (1998). Perceptual learning reflects external noise filtering and internal noise reduction through channel reweighting. *Proc. Natl. Acad. Sci. U.S.A.* 95 13988–13993. 10.1073/pnas.95.23.13988 9811913 PMC25004

[B18] Ecker A. S. Berens P. Cotton R. J. Subramaniyan M. Denfield G. H. Cadwell C. R. (2014). State dependence of noise correlations in macaque primary visual cortex. *Neuron* 82 235–248. 10.1016/j.neuron.2014.02.006 24698278 PMC3990250

[B19] Engel T. A. Steinmetz N. A. Gieselmann M. A. Thiele A. Moore T. Boahen K. (2016). Selective modulation of cortical state during spatial attention. *Science* 354 1140–1144. 10.1126/science.aag1420 27934763

[B20] Freyer F. Becker R. Dinse H. R. Ritter P. (2013). State-dependent perceptual learning. *J. Neurosci.* 33 2900–2907. 10.1523/JNEUROSCI.4039-12.2013 23407948 PMC6619196

[B21] Fries P. Reynolds J. H. Rorie A. E. Desimone R. (2001). Modulation of oscillatory neuronal synchronization by selective visual attention. *Science* 291 1560–1563. 10.1126/science.291.5508.1560 11222864

[B22] Gervasoni D. Lin S. C. Ribeiro S. Soares E. S. Pantoja J. Nicolelis M. A. (2004). Global forebrain dynamics predict rat behavioral states and their transitions. *J. Neurosci.* 24 11137–11147. 10.1523/JNEUROSCI.3524-04.2004 15590930 PMC6730270

[B23] Ghose G. M. Yang T. Maunsell J. H. (2002). Physiological correlates of perceptual learning in monkey V1 and V2. *J. Neurophysiol.* 87 1867–1888. 10.1152/jn.00690.2001 11929908

[B24] Gilbert C. D. Sigman M. (2007). Brain states: top-down influences in sensory processing. *Neuron* 54 677–696. 10.1016/j.neuron.2007.05.019 17553419

[B25] Goard M. Dan Y. (2009). Basal forebrain activation enhances cortical coding of natural scenes. *Nat. Neurosci.* 12 1444–1449. 10.1038/nn.2402 19801988 PMC3576925

[B26] Gregoriou G. G. Gotts S. J. Zhou H. Desimone R. (2009). High-frequency, long-range coupling between prefrontal and visual cortex during attention. *Science* 324 1207–1210. 10.1126/science.1171402 19478185 PMC2849291

[B27] Gutnisky D. A. Beaman C. Lew S. E. Dragoi V. (2017). Cortical response states for enhanced sensory discrimination. *eLife* 6:e29226. 10.7554/eLife.29226 29274146 PMC5760207

[B28] Harris K. D. Thiele A. (2011). Cortical state and attention. *Nat. Rev. Neurosci.* 12 509–523. 10.1038/nrn3084 21829219 PMC3324821

[B29] Kanashiro T. Ocker G. K. Cohen M. R. Doiron B. (2017). Attentional modulation of neuronal variability in circuit models of cortex. *eLife* 6:e23978. 10.7554/eLife.23978 28590902 PMC5476447

[B30] Kwon S. E. Yang H. Minamisawa G. O’Connor D. H. (2016). Sensory and decision-related activity propagate in a cortical feedback loop during touch perception. *Nat. Neurosci.* 19 1243–1249. 10.1038/nn.4356 27437910 PMC5003632

[B31] Law C. T. Gold J. I. (2008). Neural correlates of perceptual learning in a sensory-motor, but not a sensory, cortical area. *Nat. Neurosci.* 11 505–513. 10.1038/nn2070 18327253 PMC2424192

[B32] Law C. T. Gold J. I. (2009). Reinforcement learning can account for associative and perceptual learning on a visual-decision task. *Nat. Neurosci.* 12 655–663. 10.1038/nn.2304 19377473 PMC2674144

[B33] Lee S. Kruglikov I. Huang Z. J. Fishell G. Rudy B. (2013). A disinhibitory circuit mediates motor integration in the somatosensory cortex. *Nat. Neurosci.* 16 1662–1670. 10.1038/nn.3544 24097044 PMC4100076

[B34] Letzkus J. J. Wolff S. B. Meyer E. M. Tovote P. Courtin J. Herry C. (2011). A disinhibitory microcircuit for associative fear learning in the auditory cortex. *Nature* 480 331–335. 10.1038/nature10674 22158104

[B35] Li W. Piech V. Gilbert C. D. (2004). Perceptual learning and top-down influences in primary visual cortex. *Nat. Neurosci.* 7 651–657. 10.1038/nn1255 15156149 PMC1440483

[B36] Luck S. J. Chelazzi L. Hillyard S. A. Desimone R. (1997). Neural mechanisms of spatial selective attention in areas V1, V2, and V4 of macaque visual cortex. *J. Neurophysiol.* 77 24–42. 10.1152/jn.1997.77.1.24 9120566

[B37] Luczak A. Bartho P. Harris K. D. (2013). Gating of sensory input by spontaneous cortical activity. *J. Neurosci.* 33 1684–1695. 10.1523/JNEUROSCI.2928-12.2013 23345241 PMC3672963

[B38] Makino H. Komiyama T. (2015). Learning enhances the relative impact of top-down processing in the visual cortex. *Nat. Neurosci.* 18 1116–1122. 10.1038/nn.4061 26167904 PMC4523093

[B39] Marguet S. L. Harris K. D. (2011). State-dependent representation of amplitude-modulated noise stimuli in rat auditory cortex. *J. Neurosci.* 31 6414–6420. 10.1523/JNEUROSCI.5773-10.2011 21525282 PMC3099304

[B40] McGinley M. J. David S. V. McCormick D. A. (2015a). Cortical membrane potential signature of optimal states for sensory signal detection. *Neuron* 87 179–192. 10.1016/j.neuron.2015.05.038 26074005 PMC4631312

[B41] McGinley M. J. Vinck M. Reimer J. Batista-Brito R. Zagha E. Cadwell C. R. (2015b). Waking state: rapid variations modulate neural and behavioral responses. *Neuron* 87 1143–1161. 10.1016/j.neuron.2015.09.012 26402600 PMC4718218

[B42] Mitchell J. F. Sundberg K. A. Reynolds J. H. (2009). Spatial attention decorrelates intrinsic activity fluctuations in macaque area V4. *Neuron* 63 879–888. 10.1016/j.neuron.2009.09.013 19778515 PMC2765230

[B43] Ni A. M. Ruff D. A. Alberts J. J. Symmonds J. Cohen M. R. (2018). Learning and attention reveal a general relationship between population activity and behavior. *Science* 359 463–465. 10.1126/science.aao0284 29371470 PMC6571104

[B44] Otazu G. H. Tai L. H. Yang Y. Zador A. M. (2009). Engaging in an auditory task suppresses responses in auditory cortex. *Nat. Neurosci.* 12 646–654. 10.1038/nn.2306 19363491 PMC4084972

[B45] Pachitariu M. Lyamzin D. R. Sahani M. Lesica N. A. (2015). State-dependent population coding in primary auditory cortex. *J. Neurosci.* 35 2058–2073. 10.1523/JNEUROSCI.3318-14.2015 25653363 PMC4315834

[B46] Pi H. J. Hangya B. Kvitsiani D. Sanders J. I. Huang Z. J. Kepecs A. (2013). Cortical interneurons that specialize in disinhibitory control. *Nature* 503 521–524. 10.1038/nature12676 24097352 PMC4017628

[B47] Poulet J. F. Petersen C. C. (2008). Internal brain state regulates membrane potential synchrony in barrel cortex of behaving mice. *Nature* 454 881–885. 10.1038/nature07150 18633351

[B48] Rabinowitz N. C. Goris R. L. Cohen M. Simoncelli E. P. (2015). Attention stabilizes the shared gain of V4 populations. *eLife* 4:e08998. 10.7554/eLife.08998 26523390 PMC4758958

[B49] Recanzone G. H. Schreiner C. E. Merzenich M. M. (1993). Plasticity in the frequency representation of primary auditory cortex following discrimination training in adult owl monkeys. *J. Neurosci.* 13 87–103. 10.1523/JNEUROSCI.13-01-00087.1993 8423485 PMC6576321

[B50] Reimer J. Froudarakis E. Cadwell C. R. Yatsenko D. Denfield G. H. Tolias A. S. (2014). Pupil fluctuations track fast switching of cortical states during quiet wakefulness. *Neuron* 84 355–362. 10.1016/j.neuron.2014.09.033 25374359 PMC4323337

[B51] Renart A. de la Rocha J. Bartho P. Hollender L. Parga N. Reyes A. (2010). The asynchronous state in cortical circuits. *Science* 327 587–590. 10.1126/science.1179850 20110507 PMC2861483

[B52] Roth M. M. Dahmen J. C. Muir D. R. Imhof F. Martini F. J. Hofer S. B. (2016). Thalamic nuclei convey diverse contextual information to layer 1 of visual cortex. *Nat. Neurosci.* 19 299–307. 10.1038/nn.4197 26691828 PMC5480596

[B53] Sachidhanandam S. Sreenivasan V. Kyriakatos A. Kremer Y. Petersen C. C. (2013). Membrane potential correlates of sensory perception in mouse barrel cortex. *Nat. Neurosci.* 16 1671–1677. 10.1038/nn.3532 24097038

[B54] Scholvinck M. L. Saleem A. B. Benucci A. Harris K. D. Carandini M. (2015). Cortical state determines global variability and correlations in visual cortex. *J. Neurosci.* 35 170–178. 10.1523/JNEUROSCI.4994-13.2015 25568112 PMC4287140

[B55] Schoups A. Vogels R. Qian N. Orban G. (2001). Practising orientation identification improves orientation coding in V1 neurons. *Nature* 412 549–553. 10.1038/35087601 11484056

[B56] Seitz A. Watanabe T. (2005). A unified model for perceptual learning. *Trends Cogn. Sci.* 9 329–334. 10.1016/j.tics.2005.05.010 15955722

[B57] Shadlen M. N. Britten K. H. Newsome W. T. Movshon J. A. (1996). A computational analysis of the relationship between neuronal and behavioral responses to visual motion. *J. Neurosci.* 16 1486–1510. 10.1523/JNEUROSCI.16-04-01486.1996 8778300 PMC6578557

[B58] Steriade M. Timofeev I. Grenier F. (2001). Natural waking and sleep states: a view from inside neocortical neurons. *J. Neurophysiol.* 85 1969–1985. 10.1152/jn.2001.85.5.1969 11353014

[B59] Tan A. Y. Chen Y. Scholl B. Seidemann E. Priebe N. J. (2014). Sensory stimulation shifts visual cortex from synchronous to asynchronous states. *Nature* 509 226–229. 10.1038/nature13159 24695217 PMC4067243

[B60] Tetzlaff T. Helias M. Einevoll G. T. Diesmann M. (2012). Decorrelation of neural-network activity by inhibitory feedback. *PLoS Comput. Biol.* 8:e1002596. 10.1371/journal.pcbi.1002596 23133368 PMC3487539

[B61] Vinck M. Batista-Brito R. Knoblich U. Cardin J. A. (2015). Arousal and locomotion make distinct contributions to cortical activity patterns and visual encoding. *Neuron* 86 740–754. 10.1016/j.neuron.2015.03.028 25892300 PMC4425590

[B62] Watanabe T. Sasaki Y. (2015). Perceptual learning: toward a comprehensive theory. *Annu. Rev. Psychol.* 66 197–221. 10.1146/annurev-psych-010814-015214 25251494 PMC4286445

[B63] Xiao L. Q. Zhang J. Y. Wang R. Klein S. A. Levi D. M. Yu C. (2008). Complete transfer of perceptual learning across retinal locations enabled by double training. *Curr. Biol.* 18 1922–1926. 10.1016/j.cub.2008.10.030 19062277 PMC3045109

[B64] Yang T. Maunsell J. H. (2004). The effect of perceptual learning on neuronal responses in monkey visual area V4. *J. Neurosci.* 24 1617–1626. 10.1523/JNEUROSCI.4442-03.2004 14973244 PMC6730469

[B65] Zhang J. Y. Zhang G. L. Xiao L. Q. Klein S. A. Levi D. M. Yu C. (2010). Rule-based learning explains visual perceptual learning and its specificity and transfer. *J. Neurosci.* 30 12323–12328. 10.1523/JNEUROSCI.0704-10.2010 20844128 PMC3842491

[B66] Zhang S. Xu M. Kamigaki T. Hoang Do J. P. Chang W. C. Jenvay S. (2014). Selective attention. Long-range and local circuits for top-down modulation of visual cortex processing. *Science* 345 660–665. 10.1126/science.1254126 25104383 PMC5776147

